# *Chironomus riparius* Proteome Responses to Spinosad Exposure

**DOI:** 10.3390/toxics8040117

**Published:** 2020-12-11

**Authors:** Hugo R. Monteiro, João L. T. Pestana, Amadeu M. V. M. Soares, Bart Devreese, Marco F. L. Lemos

**Affiliations:** 1 Department of Biology & CESAM, University of Aveiro, Campus Universitário de Santiago, 3810-193 Aveiro, Portugal; jpestana@ua.pt (J.L.T.P.); asoares@ua.pt (A.M.V.M.S.); 2 MARE—Marine and Environmental Sciences Centre, ESTM, Polytechnic of Leiria, 2520-641 Peniche, Portugal; marco.lemos@ipleiria.pt; 3 Laboratory for Microbiology, Department of Biochemistry and Microbiology, Ghent University, B-9000 Gent, Belgium; Bart.devreese@ugent.be

**Keywords:** aquatic insects, ecotoxicoproteomics, hemoglobin, iTRAQ, neurotoxic insecticides

## Abstract

The potential of proteome responses as early-warning indicators of insecticide exposure was evaluated using the non-biting midge *Chironomus riparius* (Meigen) as the model organism. *Chironomus riparius* larvae were exposed to environmentally relevant concentrations of the neurotoxic pesticide spinosad to uncover molecular events that may provide insights on the long-term individual and population level consequences. The iTRAQ labeling method was performed to quantify protein abundance changes between exposed and non-exposed organisms. Data analysis revealed a general dose-dependent decrease in the abundance of globin proteins as a result of spinosad exposure. Additionally, the downregulation of actin and a larval cuticle protein was also observed after spinosad exposure, which may be related to previously determined *C. riparius* life-history traits impairment and biochemical responses. Present results suggest that protein profile changes can be used as early warning biomarkers of pesticide exposure and may provide a better mechanistic interpretation of the toxic response of organisms, aiding in the assessment of the ecological effects of environmental contamination. This work also contributes to the understanding of the sublethal effects of insecticides in invertebrates and their molecular targets.

## 1. Introduction

The study of the impact of stressors on ecological entities is crucial for risk assessment [1]. Most often, toxicity testing is based on organism-level responses (e.g., mortality, growth, and behavior) [2]. These endpoints provide valuable and sensitive information on the organism’s performance that can be used to predict possible outcomes at the population or community levels. However, xenobiotic concentrations commonly found in the environment may not be sufficient to cause an immediate visible individual-level response [3], and when a response occurs, it may be too late to set off successful environmental management actions. In this sense, there is a need to develop new and sensitive tools that can help determine molecular initiating events that lead to adverse outcomes and thus be used as early-warning tools to predict ecological adverse effects of stressors.

The recent advances in “omic” technologies and more particularly in proteomics, enables the identification and the study of complex mixtures containing numerous proteins from a particular sample [4,5]. The application of proteomics in ecotoxicology has been expanding in recent years, and while initially most aquatic toxicity studies focused on fish species [6], recent research has been published on aquatic invertebrates [7,8,9,10,11]. With the development of methodologies such as iTRAQ (isobaric tags for relative and absolute quantitation), it is now possible to simultaneously analyze and relatively quantify proteins from up to eight different samples, a significant advantage in comparison with traditional gel-based techniques such as two-dimensional difference gel electrophoresis (2D-DIGE) [12,13]. Studying the interaction of a specific chemical with an organism at a molecular level can lead to the discovery of potential biomarkers of effect and a better interpretation of its primary and secondary mechanisms of action within the organism [6,12,14,15,16,17].

Chironomids have a wide distribution around the globe and are frequently the most abundant group within freshwater benthic invertebrate communities [18,19,20,21]. From an ecotoxicological point of view, chironomids exhibit additional interesting features making them model organisms for acute and chronic toxicity tests as they: (1) have a short life cycle and are relatively easy to culture and handle in a laboratory; (2) live in a water-sediment interface; (3) have an essential role in organic recycling and are important prey items for different predators; and (4) usually are not target species for pesticide application [19,20,22]. Additionally, from an (ecotoxico)proteomic perspective, chironomids complex life-cycle, which includes a complete metamorphosis [22], and the fact that many species possess hemoglobin in their larval stages are also aspects of interest [23,24,25]. Nonetheless and to the best of our knowledge, studies of protein expression changes in *C. riparius* are limited to the works by Lee et al. [26] and by Choi and Ha [27], who assessed the changes in protein expression after exposure to cadmium and by Monteiro et al. [28] using the insecticide fipronil.

Spinosad is an insecticide that acts as a nicotinic acetylcholine receptor allosteric modulator [29]. Although it is registered for agricultural use, there is still limited information on the effects of this chemical on non-target aquatic invertebrates and its secondary molecular targets. Nonetheless, spinosad exposure has been shown to impair the growth and developmental rates of *C. riparius* [30]. In the present study, the effects of spinosad in *C. riparius* protein abundance profiles are evaluated in order to have a more accurate understanding of the affected biologic pathways underlying higher-level responses previously observed under exposure to this insecticide, using a similar concentration range.

## 2. Material and Methods

### 2.1. Test Chemicals

Spinosad (CAS number 168316-95-8) was acquired from Sigma-Aldrich, Gillingham, UK. To prepare working and experimental solutions, stock solutions (prepared in ethanol) were diluted with American Society for Testing and Materials (ASTM) hard water [31] and the final solvent concentration was kept at 0.01% in all experimental solutions.

### 2.2. Organism Culture and Exposure

*Chironomus riparius* egg masses were collected from a laboratory culture maintained at the University of Aveiro, Portugal. After hatching, larvae were kept in plastic aquaria filled with ASTM and a layer of commercial sterilized sand (<1 mm) at 16:8 h light: dark cycle, and fed with macerated fish food (Tetramin^®^) until reaching the desired age (8 days old). Larvae were then transferred to glass crystalizing dishes (10.7 cm base diameter) with 200 mL of spinosad (0, 0.5, 2, and 8 μg L^−1^) solution. Four replicates were used per treatment, and each replicate consisted of 20 larvae. After 48 h of exposure, all larvae from each replicate were collected and transferred to a microtube, and immediately frozen in liquid nitrogen and stored at −80 °C until further use. For both the culture and experiment, the temperature was set at 20 ± 1 °C.

The present experiment was designed assuming that changes at the molecular level precede the effects at the organism level, and these changes may be assessed earlier and at lower concentrations [17]. In this sense, the concentrations used in this study were selected based on previous experiments: two concentrations that did not cause observable long-term effects on *C. riparius* larvae development (0.5 and 2 μg L^−1^; exposure of first instar larvae), and the lowest observable effect concentration at organismal level (8 μg L^−1^), for which changes in growth, emergence, and development were observed [30]. Additionally, the use of environmentally relevant concentrations was also a major aim of this study.

### 2.3. Protein Extraction

Protein extraction was performed following a TCA-acetone extraction method as described by Cilia et al. [32] with minor modifications. Briefly, samples were homogenized with a mechanical homogenizer (Ystral d-7801, Ballrechten-Dottingen, Germany). A few microliters of K-phosphate buffer 0.1 M were added to each sample, to aid the homogenization process. Samples were then gently mixed with 10% trichloroacetic acid (TCA) in acetone containing 2% β-mercaptoethanol (10 mL TCA-Acetone per g of sample). After overnight incubation at −20 °C, samples were centrifuged at 5000× *g* during 30 min and the pellets formed were washed in acetone. These two steps were repeated until tissue debris was completely discarded. Acetone used in this protocol was previously stored at −20 °C, and homogenization and extraction steps were performed on ice. The resulting pellets were solubilized in a 0.04 M Tris-HCl buffer solution at pH = 8 with 7 M urea, 2 M thiourea, 0.05 M MgCl_2_, 0.5% Triton-X-100, and 0.1% SDS. To prevent protein degradation, a protease inhibitor cocktail (Roche, Mannheim, Germany), 1% bovine pancreas DNase I (Roche, Mannheim, Germany), and 1% bovine pancreas RNase A (Roche, Mannheim, Germany) were added to the extracts and stored at −80 °C until further use.

### 2.4. Sample Preparation for iTRAQ^®^

Two iTRAQ 8plex runs were made, each run consisting of two biological replicates of each treatment (giving a total of four biological replicates per treatment). To remove potential interfering compounds with iTRAQ labeling, an acetone precipitation was performed according to the manufacturer instructions (iTRAQ Reagents–8plex protocol; AB Sciex, Framingham, MA, USA) and proteins were resuspended in 0.5 M triethylammonium bicarbonate (TEAB) buffer. Protein content was determined using Coomassie Plus™ Kit assay (Thermo Fisher Scientific, Waltham, MA, USA), and 5 μg of each sample was loaded onto an SDS-Page gel to verify extraction efficiency and integrity of proteins. Afterwards, 20 μg of each sample was separated, dried in a SpeedVac™ (SC110; Thermo Savant, Holbrook, NY, USA), and resuspended in a total volume of 25 µL of 0.5 M TEAB buffer to initiate iTRAQ labeling protocol. Succinctly, 1 μL of denaturant and 2 μL of reducing agents provided with the kit were added to the sample and incubated at 60 °C for 1 h. Afterwards, 1 μL of cysteine blocking reagent was added. Samples were incubated for 10 min at room temperature, followed by the addition of 10 μL of TEAB buffer. After overnight trypsin digestion (trypsin:protein ratio of 1:50, Sequencing Grade Modified Trypsin, Promega, Madison, WI, USA), resulting peptides were labeled as shown in Table 1 and pooled. Before advancing to the separation of peptides, labeling efficiency was checked by MS/MS, and 1 μL of each sample was cleaned using Agilent Bond Elut OMIX C18 tips according to manufacturer’s guidelines but using 0.1% trifluoroacetic acid (TFA) as washing solution. After pooling, samples were dried and stored at −20 °C.

### 2.5. Two-Dimensional Reversed Phase Liquid Chromatography

To reduce complexity, a fractionation of samples was made using a two-dimensional high-performance liquid chromatography (2D-HPLC) approach, specifically a high-pH/low-pH reversed phase (RP) liquid chromatography. This separation method was proposed by Gilar et al. [33] and has been successfully used in combination with iTRAQ [34]. The first dimension (at high pH) was performed in a ETTAN LC chromatograph (GE Healthcare, Buckinghamshire, UK) using a Gemini^®^ C18 LC Column (100 mm × 1 mm, 3 µm, 110 Å; Phenomenex, Torrance, CA, USA) as the stationary phase while 2% acetonitrile (ACN), 0.02 M ammonium formate, pH = 10 (Buffer A1) and 80% ACN, 0.02 M ammonium formate, pH = 10 (Buffer B1) were used as mobile phases with a flow of 0.05 mL min^−1^. A total of 100 μg of peptides previously diluted in buffer A1 were injected in each run. The gradient employed was as follows: starting with 5 min of 100% buffer A1, it was followed by a 30-min linear increase of 0–50% buffer B1 and then a linear increase from 50 to 100% buffer B1 for 1 min. The separation gradient remained at 100% for 6 min before ending the run with a 7-min 100% buffer A1. The eluted peptides were monitored at 214, 220, and 280 nm and collected to 8 different fractions for each run. After collection, samples were dried, resuspended in a 2% ACN and 0.1% TFA solution and stored at −20 °C when not immediately injected in the second-dimension chromatograph.

The second RP-LC (low pH) was performed in a Dionex™ LC Packings system equipped with a Famos™ autosampler, a Switchos™ switching unit (with a loading pump), an Ultimate™ dual gradient system, and a Probot™ spotting device. Five microliters of each sample were first injected and concentrated in an Acclaim™ PepMap™ C18 trapping column (0.3 mm × 5 mm, 5 µm, 100 Å) using 2% ACN and 0.1% formic acid as the mobile phase at a flow of 0.025 mL min^−1^. After 5 min, samples were eluted onto the analytical column Acclaim PepMap C18 nanoviper (0.075 mm × 150 mm, 3 µm, 100 Å). The eluents used for peptide separation were 100% H_2_0, 0.1% TFA (Buffer A2), 100% ACN, and 0.1% TFA (Buffer B2). The gradient employed was as follows: 3 min of 1% B2, followed by a 25-min linear increase to 50% B2, and a subsequent a linear increase from 50% to 100% B2 for 10 min; the gradient remained at 100% B2 for 5 min before returning to the initial settings (1% B2). The pump flow was set at 0.3 μL min^−1^. Eluted peptides were monitored at 214 and 280 nm using an Ultimate™ UV Detector, and at 3 min into the run, Probot spotting device was turned on and started spotting the samples onto an Opti-TOF™ LC MALDI plate every 30 s. Spotted samples were promptly manually mixed with a supporting matrix, consisting of 4 mg ml^−1^ of α-Cyano-4-hydroxycinnamic acid, 70% ACN, 0.01 M dibasic ammonium citrate, and 0.1% TFA.

### 2.6. Mass Spectrometric Analysis, Protein Identification, and Quantification

Mass spectrometric analysis was performed using a 4800 Plus MALDI TOF/TOF Analyzer system (AB Sciex, Framingham, MA, USA). MS spectra were acquired using the positive ion reflector mode and the six most intense peaks (minimum S/N ratio of 15) were selected for MS/MS peptide fragmentation.

All MS/MS data retrieved were processed using ProteinPilot™ software v. 4.0. This software allows the inference of proteins by the identification of peptides using the Paragon™ algorithm (AB Sciex, Framingham, MA, USA) [35], and also the relative quantification of iTRAQ labeled peptides. The following parameters were applied for the analysis: iTRAQ 8 plex (peptide labeled); MMTS (methyl methanethiosulfonate) was set as the cysteine-blocking reagent used during peptide labeling; digestion with trypsin; and MALDI 4800 as the instrument used. Variable biological modifications and amino acid substitutions were checked for ID purposes. Concerning the quantification analysis, background and bias corrections were applied. All the datasets were searched against a database resulting from the translated transcriptome of *C. riparius* [36]. Transcripts were obtained from NCBI Transcriptome Shotgun Assembly (TSA) database (Bioproject PRJNA167567) [36] and translated using the OrfPredictor tool [37]. To this database, a list of contaminant proteins provided with the software and a (reversed) decoy database were used to reduce false positive peptide hits. Since this database may not cover the full transcriptome of *C. riparius*, datasets were also blasted against a database of dipteran proteins deposited on NCBI using the same settings. Positive matches on this database were manually inspected to discard duplicate protein hits. Translated protein hits were searched using the NCBI BLASTX tool against non-redundant protein sequences database and the top result was annotated. For quantification analysis, only hits within 5% false discovery rate (FDR) and with an “unused score” greater than 1 (90% confidence) were considered. Since absolute quantification by iTRAQ would require the use of a standard in each run [38], and that would be very limiting in terms of the experimental design, samples were normalized to one of the control replicates, and average protein ratios determined were used for statistical analysis.

### 2.7. Statistical Analysis

A linear mixed model was used to determine changes in the expression of each protein between experimental treatments and the control treatment. Treatment (treated as a categorical variable) was set as fixed factor, while iTRAQ run was set as a random factor to account for variability amongst runs. When the random effect variance was estimated to be zero, a simpler model without the random factor was used instead. All data were checked for normality using residual plots. When necessary, data were transformed using a log transformation to meet the assumption of normality. Only proteins identified and with average protein ratios determined in both runs, were used. Linear mixed model analysis was performed using IBM SPSS^®^ 25 for Mac. A linear regression was used to assess the relationship between spinosad concentration and globin expression using GraphPad Prism^®^ 7 for Mac. The significance level was set at *p* < 0.05 for all statistical tests.

## 3. Results

**Figure 1:**
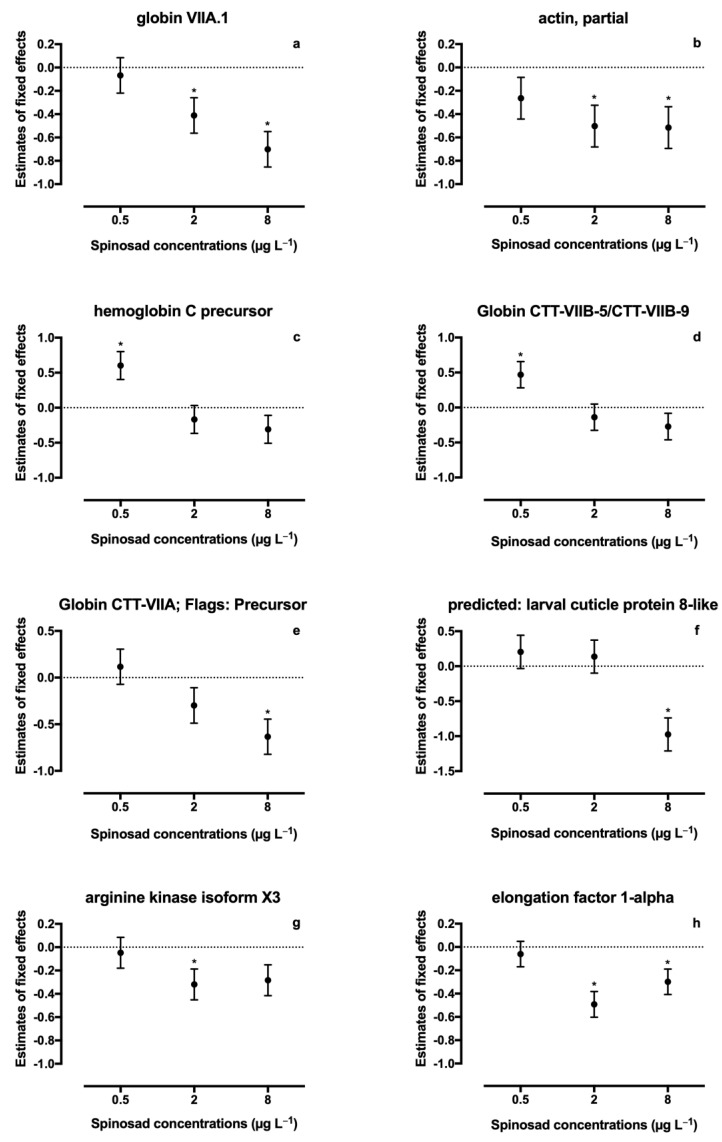
Changes in proteins abundance in *Chironomus riparius* after exposure to Spinosad. Plots are presented as estimates of fixed effects ± SE from linear mixed model. Asterisks indicate significant differences; (**a**) globin VIIA.1; (**b**) actin, partial; (**c**) hemoglobin C precursor; (**d**) globin CTT-VIIB-5/CTT-VIIB-9; (**e**) globin CTT-VIIA; flags: precursor; (**f**) predicted: larval cuticle protein 8-like; (**g**) arginine kinase isoform X3; and (**h**) elongation factor 1-alpha.

**Figure 2:**
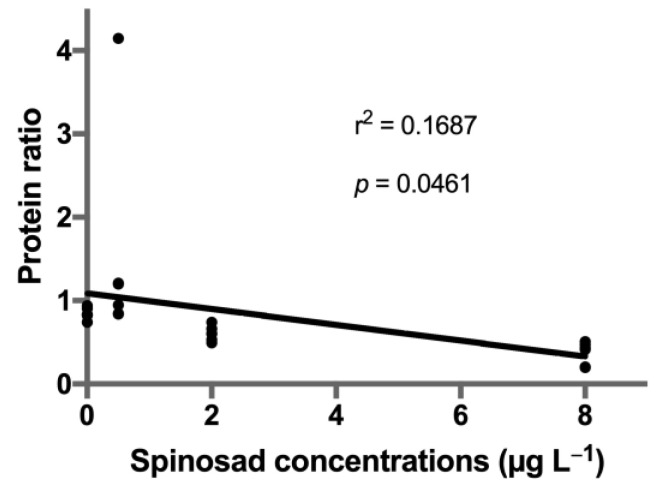
Linear regression of the ratios of identified globins expression in *Chironomus riparius* after exposure to spinosad. *p* value indicates deviations from zero slope.

A total of thirty-six proteins were identified in spinosad-exposed *C. riparius* larvae (Table S1). From these, fifteen proteins identified in both iTRAQ runs were considered for quantification analysis, and eight proteins (22.2%) were found to be differentially expressed (Table 2, Figure 1): four proteins belonging to the globin family, one cuticle protein, one actin, one arginine kinase, and one elongation factor. A significant decrease in expression of protein KA196492 (Globin CTT-VIIA) was observed in the 8 μg L^−1^ treatment (estimate ± SE = −0.633 ± 0.190, t = −3.330, *p* = 0.006). A similar response was observed for protein KA195409 (larval cuticle protein), which was also underexpressed at 8 μg L^−1^ of spinosad (estimate ± SE = −0.974 ± 0.237, t = −4.112, *p* = 0.001). Protein KA178027 (arginine kinase) was significantly downregulated at 2 μg L^−1^ of spinosad (estimate ± SE = −0.319 ± 0.133, t = −2.387, *p* = 0.034) and, although not significant, a decrease in the expression was noticeable in the highest concentration tested (estimate ± SE = −0.283 ± 0.133, t = −2.124, *p* = 0.055). Protein KA181893 (Globin VIIA.1) expression decreased in the two highest concentrations tested (2 μg L^−1^ treatment: estimate ± SE = −0.411 ± 0.152, t = −2.699, *p* = 0.019; 8 μg L^−1^ treatment: estimate ± SE = −0.701 ± 0.152, t = −4.603, *p* = 0.001). A decrease in the two highest concentrations tested was also observed for proteins KA184259 (actin; 2 μg L^−1^ treatment: estimate ± SE = −0.503 ± 0.179, t = −2.809, *p* = 0.017; 8 μg L^−1^ treatment: estimate ± SE = −0.515 ± 0.179, t = −2.879, *p* = 0.015) and KA183802 (elongation factor 1-alpha; 2 μg L^−1^ treatment: estimate ± SE = −0.492 ± 0.110, t = −4.473, *p* = 0.001; 8 μg L^−1^ treatment: estimate ± SE = −0.299 ± 0.110, t = −2.717, *p* = 0.020). Proteins KA177778 (hemoglobin C precursor; estimate ± SE = 0.602 ± 0.200, t = 3.013, *p* = 0.012) and KA193165 (Globin CTT-VIIB-5/CTT-VIIB-9; estimate ± SE = 0.470 ± 0.189, t = 2.479, *p* = 0.031) abundance increased in the 0.5 μg L^−1^ treatment. A significant linear regression was found between spinosad concentration and decreased expression levels of all six identified globins (r^2^ = 0.17, *p* = 0.046; Figure 2).

## 4. Discussion

The present study shows that exposure to spinosad can cause alterations in the proteome of *C. riparius*. The changes in protein expression observed here for globins, actin, cuticle proteins, arginine kinase, and elongation factor 1-alpha can aid to understand the mechanisms involved in spinosad’s toxic action and reveal indirect effects that, together with its neurotoxic mode action, may contribute to the responses seen at higher levels of biological organization.

An overall analysis of the data revealed that globins, in general, decreased as the concentration of the pesticide increased. The function of hemoglobins (Hbs) in *Chironomus* sp. and their ecotoxicological relevance have been extensively studied. These are the most abundant proteins in *C. riparius* larvae [39]. Hemoglobins perform a respiratory function in *Chironomus*, and due to their high affinity for oxygen [23,40,41] they are capable of maintaining a good oxygen supply for aerobic metabolism even under hypoxic conditions [23,42]. It is therefore postulated that freshwater invertebrates containing high amounts of Hb are very tolerant to adverse environmental conditions [23,27,43], and hemoglobins have been previously proposed as potential biomarkers for environmental monitoring [24,27,44,45,46]. The downregulation of these proteins may increase the vulnerability of *C. riparius* larvae to chemical stress and has been associated with decreased growth and development. Choi and Ha (2009) [27] reported a generalized decrease in the expression of globins (and subsequently a decrease of total Hb content) as a consequence of exposure to cadmium. These authors also observed a decreased larval weight together with a decrease in emergence and reproductive traits, and conjectured that these outcomes may be directly related to the alterations of globins expression. The impairment of larval growth and emergence of *C. riparius* by spinosad exposure was previously observed [30], suggesting that these outcomes are not exclusively associated with the neuromuscular toxicity of spinosad, but are also associated with the underexpression of globin proteins. Moreover, previous studies indicated that these concentrations of spinosad increased the electron transport system (ETS) activity of *C. riparius*, denoting higher cellular oxygen consumption [30]. This increased ETS activity accompanied by the downregulation of globin proteins may result in a deficient oxygen supply to cells and tissues, which may, on the long-term, lead to hypoxia and contribute to the previously observed chronic effects at the individual level [30]. This action on globins expression may have also caused the larvae to switch to anaerobic metabolism and become more dormant [18], which is supported by an increase in lactate dehydrogenase activity previously observed under spinosad exposure [30].

A decline of Hb production in *Chironomus* larvae is only expected to occur during molting periods [23,47,48]. This decline observed during the intermolt period suggests that globins may be directly affected by spinosad exposure. Interestingly, for two globins identified, there was an increase in their expression in the lowest concentration tested, with a fourfold increase observed for one of these proteins (KA177778). This induction at low concentrations may be associated with hemoglobin roles in oxygen transportation and storage, providing a good oxygen supply for oxygen-dependent detoxification mechanisms [27,49]. Moreover, a possible direct role of Hbs in the detoxification of xenobiotics has been suggested [23,49]. Despite this increase observed in the lower concentration, expression levels of these proteins in the two highest concentrations decreased to lower levels than those observed for non-exposed organisms, similar to the other identified globins’ effects. This suggests that at higher concentrations, spinosad’s toxicity may be systemic and affect larval response mechanisms.

A significant decrease was also detected for actin. Actin is one of the most abundant proteins in eukaryotic cells [50,51]. This cytoskeleton protein is involved in many physiological processes, including cellular motility, muscle contraction, and cytokinesis [50,52,53,54]. Several studies have reported alterations of actin state due to oxidative damage [55,56,57,58]. A decrease in the expression of *C. riparius* actin as a response to cadmium contamination has been previously observed [26], and the authors suggested a possible association between this decrease and the behavioral changes observed (decreased case-building ability). In this study, behavioral endpoints were not directly assessed, nonetheless changes in growth and survival of the larvae were previously observed for spinosad exposure, and evidence of oxidative damage (e.g., increased lipid peroxidation) [30]. The decrease observed here in actin expression may therefore reflect the spinosad-induced neuromuscular toxicity and oxidative stress on *C. riparius*. The downregulation of actin and globin proteins was previously observed in *C. riparius* under exposure to fipronil (also a neurotoxic insecticide) [28], suggesting that these proteins, although not specific, may be candidate biomarkers of insecticide exposure in *C. riparius* larvae.

A significant decrease in a cuticle protein was also observed in larvae exposed to spinosad. Insect cuticle is composed of cuticular proteins and chitin, key components of insect exoskeleton and crucial for molting and development [59]. Although cuticular penetration of spinosad is expected to be relatively slow [29], alterations on the arthropod *Blattella germanica* cuticle hydrocarbon profile due to spinosad exposure have been reported before [60]. The downregulation of cuticle proteins may interfere with cuticle permeability and molting and consequently with the growth and reproduction of arthropods [61]. Since chironomids’ growth, molting, metamorphosis, and other life traits are controlled by hormones [22,62], it is of interest to note that the proteins examined above are, to a certain extent, regulated by hormones [49,63,64]. The possibility of spinosad interacting with growth hormones cannot be inferred from this study, and there is no reported evidence of spinosad’s endocrine disrupting effects [65,66]. Nonetheless, more research should be conducted to elucidate if the downregulation of these proteins is a direct effect of the pesticide or if spinosad has an endocrine disrupting activity on *C. riparius*—in any of these cases, hormone direct or indirect impairment may bring up other effects at higher levels of biological organization as these messengers are the cornerstone molecules of a myriad of major biological processes such as growth and reproduction.

Other proteins found to be differentially expressed after exposure to spinosad were arginine kinase isoform X3 and elongation factor 1-alpha (Table 2). Arginine kinase (AK) is involved in the cellular energy metabolism of invertebrates, catalyzing the reversible conversion of L-arginine and adenosine triphosphate (ATP) to phosphoarginine and adenosine diphosphate (ADP) [11,67]. The observed downregulation of AK suggests, again, disturbance of the energy metabolism caused by spinosad exposure. Nevertheless, a significant change in AK expression was only observed in the 2 μg L^−1^ treatment, despite a decrease being also observed in the 8 μg L^−1^ treatment. A decrease in AK expression has been reported in dipterans under xenobiotic stress [68,69]. Decreases in the expression of elongation factor 1-alpha at the two highest tested concentrations suggest some disturbance on the protein biosynthesis. This is in line with the other changes observed here—all proteins differentially expressed at these concentrations were downregulated.

A non-monotonic response was observed for some proteins—the expression of two globins significantly increased in the lowest tested concentration and arginine kinase was only significantly downregulated at the 2 μg L^−1^. These responses underline the importance of dose–response in ecotoxicoproteomic studies (including assessing effects of concentrations that produce no apparent long-term organism-level effects), since different concentrations of the same chemical can trigger different responses at the proteome level. Indeed, one of the major challenges in environmental “omics” is determining which alterations at the molecular level are responsible for the outcomes observed, and which alterations are simply unrelated, adaptive, or even beneficial [70].

In the present study, however, only a part of the complex proteome of *C. riparius* was covered, since only a few highly abundant proteins were identified. The presence of abundant proteins such as hemoglobin, which represents roughly 60% of *C. riparius* total protein content [39] or actin, which is also very abundant in eukaryotic cells, may have masked the detection of less abundant proteins, suggesting the requirement of additional sample fractionating steps when studying *C. riparius* proteome. Other relevant but less abundant proteins that were not assessed here may also have contributed to the effects observed at higher levels. This reinforces the requirement of a more integrative ecotoxicological approach, at different levels of biological organization, to uncover sensitive and early-warning protein biomarkers through a better refinement of available methods [17,71]. Another major limitation of this dataset was the use of an LC-MALDI approach to read out iTRAQ data. The use of more advanced instruments such as Orbitrap mass spectrometers coupled with iTRAQ would provide a more robust and sensitive quantification and protein coverage. Despite the changes in protein abundance observed in the present study can be associated with changes previously observed at the organism level, most of these proteins are typically considered as housekeeping proteins. This, together with the low number of identified proteins, suggest that the observations derived from this study should be further explored and validated using every time updated proteomic techniques, while still considering a range of exposure concentrations.

## 5. Conclusions

This work evaluated the effects of three concentrations of spinosad in the *C. riparius* proteome. While most ecotoxicoproteomic studies to this date focus on one single concentration of a stressor, the responses observed support the need of using techniques that allow the simultaneous analysis of several samples—especially in an era of increased awareness about non-monotonic dose–responses and its relevance when considering toxicological studies. Although the results presented in this study may be regarded as somehow limited and should be independently validated with up-to-date proteomic techniques, changes were observed at the proteome level that could be related to the effects observed at higher levels of biological organization, and be directly and/or indirectly related to insecticides’ modes of action. As suggested by other authors, globins are very promising biomarkers of stress in *C. riparius*, and the results here presented suggest that globins expression could be a potential biomarker for insecticide toxicity. iTRAQ can be a very valuable tool in ecotoxicoproteomics, since this technique allows the evaluation of dose-response relationships without disregarding the use of biological replicates. However, the experimental setup used here may still not be ideal, due to some variability between LC-MS/MS runs—only about half of the proteins identified could be further used for quantification. Despite the cost-effectiveness to a lesser investment-wise field such as ecotoxicology might be discussed, the development of higher multiplexing capacity methodologies, such as the 10-plex TMT [72], the 12-Plex DiLeu isobaric tags [73], or the 18-plex method proposed by Dephoure and Gygi [74], may be of great use in ecotoxicoproteomics. Moreover, the use of more sensitive mass spectrometers such as Orbitrap analyzers could increase the depth of analysis in terms of proteins identification, which was also a major limitation of the approach used in this study. Despite the contribution of this work to the knowledge of the effect of neurotoxic insecticides on aquatic insects, extensive research still has to be done. With the growing information and the techniques available and their costs, soon these tools will be available and adapted to rapidly screen for environmental stress and/or to uncover mechanisms of action of chemicals that are not yet known.

## Figures and Tables

**Table 1:** iTraq labeling reagents used in each run. T1, T2, and T3 refer to 0.5, 2, and 8 μg L^−1^, respectively. iTRAQ 1 refers to the first run, and ITRAQ 2 refers to the second run.

Treatment	Labeling Reagent	
	iTRAQ 1	iTRAQ 2
Control	121	116
Control	115	119
T1	116	121
T1	119	118
T2	114	115
T2	118	117
T3	117	113
T3	113	114

**Table 2:** Differentially expressed proteins in *Chironomus riparius* after exposure to spinosad.

Total Score	% Cov.	GenBank Accession #	Peptides (95%)	Blast Top Result/Protein Match	Species	Protein Accession #	Significant Changes *
20.00	59.0	KA181893	14	globin VIIA.1	*Chironomus thummi thummi*	AAB58930.1	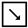 T2 and T3
18.29	47.3	KA184259	18	actin, partial	*Zygaena filipendulae*	AHW40461.1	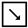 T2 and T3
18.23	89.4	KA177778	12	hemoglobin C precursor	*Chironomus thummi*	AAA28251.1	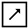 T1
16.14	78.9	KA193165	11	Globin CTT-VIIB-5/CTT-VIIB-9	*Chironomus thummi thummi*	P84298.1	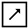 T1
14.00	72.9	KA196492	8	Globin CTT-VIIA; Flags: Precursor	*Chironomus thummi thummi*	P02226.2	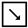 T3
12.00	82.0	KA195409	12	predicted: larval cuticle protein 8-like	*Drosophila kikkawai*	XP_017017873.1	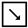 T3
8.00	24.0	KA178027	5	arginine kinase isoform X3	*Bactrocera dorsalis*	XP_029408871.1	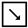 T2
4.00	14.5	KA183802	3	elongation factor 1-alpha	*Polypedilum vanderplanki*	BAH28891	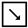 T2 and T3

Total score—ProteinPilot total score for the protein; % Cov.—The percentage of matching amino acids (of translated sequence); Peptides (95%)—The number of distinct peptides having at least 95% confidence; * T1—0.5 μg L^−1^; T2—2 μg L^−1^; T3—8 μg L^−1^; 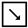 decrease; 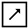 increase.

## Supplementary Materials

The following are available online at https://www.mdpi.com/2305-6304/8/4/117/s1, Table S1: Classification of proteins identified in the Spinosad exposure.
