# Supplementary material for: Chironomus riparius Proteome Responses to Spinosad Exposure
Source: Toxics. 2020 Dec 11;8(4):117. doi: 10.3390/toxics8040117 (PMC7768432; doi:10.3390/toxics8040117)
Supplement: Supplementary file 1 [file toxics-08-00117-s001.pdf]

Supplementary Materials: *Chironomus riparius* Proteome Responses to Spinosad Exposure

Hugo R. Monteiro, João L. T. Pestana, Amadeu M. V. M. Soares, Bart Devreese and Marco F. L. Lemos

Table S1. Classification of proteins identified in the spinosad exposure.

GenBank Accession #	Blast Top Result / Protein Match	Species	Protein Accession #
KA177778	hemoglobin C precursor	*Chironomus thummi*	AAA28251.1
KA181893	globin VIIA.1	*Chironomus thummi thummi*	AAB58930.1
KA193165	Globin CTT-VIIB-5/CTT-VIIB-9	*Chironomus thummi thummi*	P84298.1
KA196492	Globin CTT-VIIA	*Chironomus thummi thummi*	P02226.2
KA181976	hemoglobin A' precursor	*Chironomus thummi*	AAA28254.1
KA181871	globin 1	*Chironomus riparius*	AHV85224.1
KA183621	CLUMA_CG006317, isoform A (ATP synthase subunit beta)	*Clunio marinus*	CRK92903.1
KA185891	glyceraldehyde 3-phosphate dehydrogenase	*Haematobia irritans*	JAV18211.1
KA178027	arginine kinase isoform X3	*Daphnia magna*	JAN91448.1
KA183497	CLUMA_CG003212, isoform A (V-type proton ATPase subunit B)	*Clunio marinus*	CRK89474.1
DI336684	CLUMA_CG017016, isoform C (glutamate dehydrogenase)	*Clunio marinus*	CRL03893.1
KA184417	CLUMA_CG006885, isoform A (Enolase)	*Clunio marinus*	CRK93344.1
KA175747	CLUMA_CG009037, isoform C (Glycogenin-1)	*Clunio marinus*	CRK95573.1
KA180040	CLUMA_CG010689, isoform A (Fructose-bisphosphate aldolase)	*Clunio marinus*	CRK97294.1
KA185215	CLUMA_CG020704, isoform A (isocitrate dehydrogenase)	*Clunio marinus*	CRL07750.1
KA195409	PREDICTED: larval cuticle protein 8-like	*Drosophila kikkawai*	XP_017017873.1
KA194019	CLUMA_CG016256, isoform A (Cuticle Protein)	*Clunio marinus*	CRL02974.1
KA181949	CLUMA_CG012859, isoform A (Pupal cuticle protein)	*Clunio marinus*	CRK99541.1
KA190056	CLUMA_CG013198, isoform A (Larval cuticle protein LCP-17)	*Clunio marinus*	CRK99895.1
KA195958	CLUMA_CG016573, isoform A (Flexible cuticle protein 12)	*Clunio marinus*	CRL02972.1
KA174808	myosin heavy chain	*Anopheles darlingi*	ETN57922.1
KA184259	actin, partial	*Zygaena filipendulae*	AHW40461.1
KA180522	tubulin beta-1 chain	*Aedes albopictus*	XP_019552411.1
KA184573	tubulin alpha-1 chain-like	*Dinoponera quadriceps*	XP_019643284.1
KA180285	AGAP004877-PA (Myosin)	*Anopheles gambiae* str. PEST	XP_314309.4
KA178558	Tropomyosin-2	*Lucilia cuprina*	KNC34186.1
CAA09938	tropomyosin	*Chironomus kiiensis*	CAA09938.2
KA183802	elongation factor 1-alpha	*Culicoides sonorensis*	AAV84215.1
DI336732	CLUMA_CG016390, isoform A (40S ribosomal protein S23)	*Clunio marinus*	CRL03238.1
KA196663	CLUMA_CG017633, isoform A (40S ribosomal protein S28)	*Clunio marinus*	CRL04562.1
KA183456	heat shock cognate 70	*Chironomus yoshimatsui*	AAN14526.1
KA183270	heat shock protein 70	*Polypedilum vanderplanki*	ADM13382.1
KA183868	Plasminogen activator inhibitor 1 RNA-binding protein, partial	*Daphnia magna*	JAN92456.1

KA196010	Histone H2A	*Urechis caupo*	P27325.2
KA184744	LUMA_CG009411, isoform A (Decaprenyl-diphosphate synthase subunit 2)	*Clunio marinus*	CRK95970.1
EDW77007	GK10694 (uncharacterized protein)	*Drosophila willistoni*	EDW77007.2
